# Effects of three microtubule-associated proteins (MAP2, MAP4, and Tau) on microtubules’ physical properties and neurite morphology

**DOI:** 10.1038/s41598-023-36073-9

**Published:** 2023-05-31

**Authors:** Kohei Nishida, Kosuke Matsumura, Miki Tamura, Takuto Nakamichi, Keiya Shimamori, Masahiro Kuragano, Arif Md. Rashedul Kabir, Akira Kakugo, Susumu Kotani, Naoki Nishishita, Kiyotaka Tokuraku

**Affiliations:** 1 Graduate School of Engineering, Muroran Institute of Technology, Muroran, 050-8585 Japan; 2 Faculty of Science, Hokkaido University, Sapporo, 060-0810 Japan; 3 Department of Physics, Graduate School of Science, Kyoto University, Kyoto, 606-8502 Japan; 4 Faculty of Science, Kanagawa University, Kanagawa, 221-8686 Japan; 5 Regenerative Medicine and Cell Therapy Laboratories, Kaneka Corporation, Kobe, 650-0047 Japan

**Keywords:** Cytoskeletal proteins, Microtubules

## Abstract

The physical properties of cytoskeletal microtubules have a multifaceted effect on the expression of their cellular functions. A superfamily of microtubule-associated proteins, MAP2, MAP4, and tau, promote the polymerization of microtubules, stabilize the formed microtubules, and affect the physical properties of microtubules. Here, we show differences in the effects of these three MAPs on the physical properties of microtubules. When microtubule-binding domain fragments of MAP2, tau, and three MAP4 isoforms were added to microtubules in vitro and observed by fluorescence microscopy, tau-bound microtubules showed a straighter morphology than the microtubules bound by MAP2 and the three MAP4 isoforms. Flexural rigidity was evaluated by the shape of the teardrop pattern formed when microtubules were placed in a hydrodynamic flow, revealing that tau-bound microtubules were the least flexible. When full-length MAPs fused with EGFP were expressed in human neuroblastoma (SH-SY5Y) cells, the microtubules in apical regions of protrusions expressing tau were straighter than in cells expressing MAP2 and MAP4. On the other hand, the protrusions of tau-expressing cells had the fewest branches. These results suggest that the properties of microtubules, which are regulated by MAPs, contribute to the morphogenesis of neurites.

## Introduction

Microtubules are involved in various cellular functions such as cytokinesis, intracellular transport, and cellular morphogenesis, so their physical properties strongly influence the expression of those functions^1^. Microtubules interact with various accessory proteins and alter their physical properties. For example, end-binding 1 (EB1), a type of microtubule plus-end-tracking protein, binds to the plus end of microtubules, regulating their dynamics^2^. Katanin, a microtubule severing factor, severs and disassembles microtubules to tubulin dimers^3^. Three microtubule-associated proteins (MAPs), MAP2, MAP4, and tau^4–8^, which are the most well-studied mammalian-derived MAPs with similar primary structures, promote microtubule polymerization and stabilize formed microtubules. MAPs in the MAP2/MAP4/tau superfamily^9^ are phosphorylated by kinases such as cell division cycle protein 2 (Cdc2, also known as CDK1)^10^, protein kinase C (PKC)^11^, glycogen synthase kinase (GSK)^12^, and microtubule affinity regulating kinase (MARK)^13^, and their affinity for microtubules is regulated. Phosphorylation of these MAPs is involved in the development of Alzheimer's disease^14^ and heart failure^15^.

MAP2, MAP4, and tau are composed of a projection domain and a microtubule-binding domain (MBD)^9^. The MBD is divided into three subdomains: a Pro-rich region that is rich in proline residues, a repeat region in which the assembly-promoting sequence is tandemly repeated, and a tail region^9^. The function of MBD of the MAP2/MAP4/tau superfamily has been studied in some detail, revealing the function of each subdomain^16–22^ and their effect on microtubule-dependent motor protein movement^23–26^. MAP2, MAP4, and tau are divided into neural MAP (MAP2 and tau) and ubiquitous MAP (MAP4) based on their cellular localization^9^. It is well known that MAP2 and tau are localized in dendrites and neuronal axons of neurons, respectively^1^. MAP4 is also localized in dendrites and dendritic spines of neurons^27^. Several reports indicated that the binding of MAP2/MAP4/tau superfamily proteins altered the flexural rigidity of microtubules^18,28–30^, although their physiological role in the regulation of that flexural rigidity is not well understood. Since microtubules and actin filaments are organized in parallel within cell protrusions^31,32^, the flexural rigidity of these cytoskeletons presumably shows an effect on protrusion properties. Of note, it has been inferred that microtubules’ flexural rigidity is significantly larger than that of actin^33^, contributing significantly to the flexibility of cell protrusions. These results imply that the regulation of microtubule flexural rigidity by the MAP2/MAP4/tau superfamily has a significant effect on the formation and properties of neurites.

With the development of optical microscopes such as confocal and super-resolution microscopes, it is now possible to observe the fluctuations of individual microtubules inside and outside of cells in real time. We recently noticed, from detailed microtubule observations and using these microscopic techniques, that the flexibility of microtubules in vitro and in cells depends on the type of bound MAPs^31^. We further speculated in that study that the MAP2/MAP4/tau superfamily proteins regulate the flexibility of cell protrusions by forming microtubules with different flexibility, leading to the development of neuronal cell axons and dendrites. In this study, therefore, we attempted to quantitatively evaluate the effects of each of these three MAPs on the mechanical properties of microtubules in vitro using several methods. Furthermore, the shape of each microtubule in the protrusions of cells expressing these MAPs was analyzed at a high resolution. The results revealed that tau forms straighter and more rigid microtubules than MAP2 and MAP4. Furthermore, it was also revealed that SH-SY5Y cells expressing tau had straighter microtubules in their protrusions and formed neurites with fewer branches than cells expressing MAP2 and MAP4. These results suggest that tau is advantageous for the formation of straight protrusions without branches, such as axons, and that MAP2 and MAP4 enhance the formation of flexible protrusions with many branches, such as dendrites.

## Results

### Effect of microtubule-binding domains of MAPs on physical properties of microtubules

**Figure 1:**
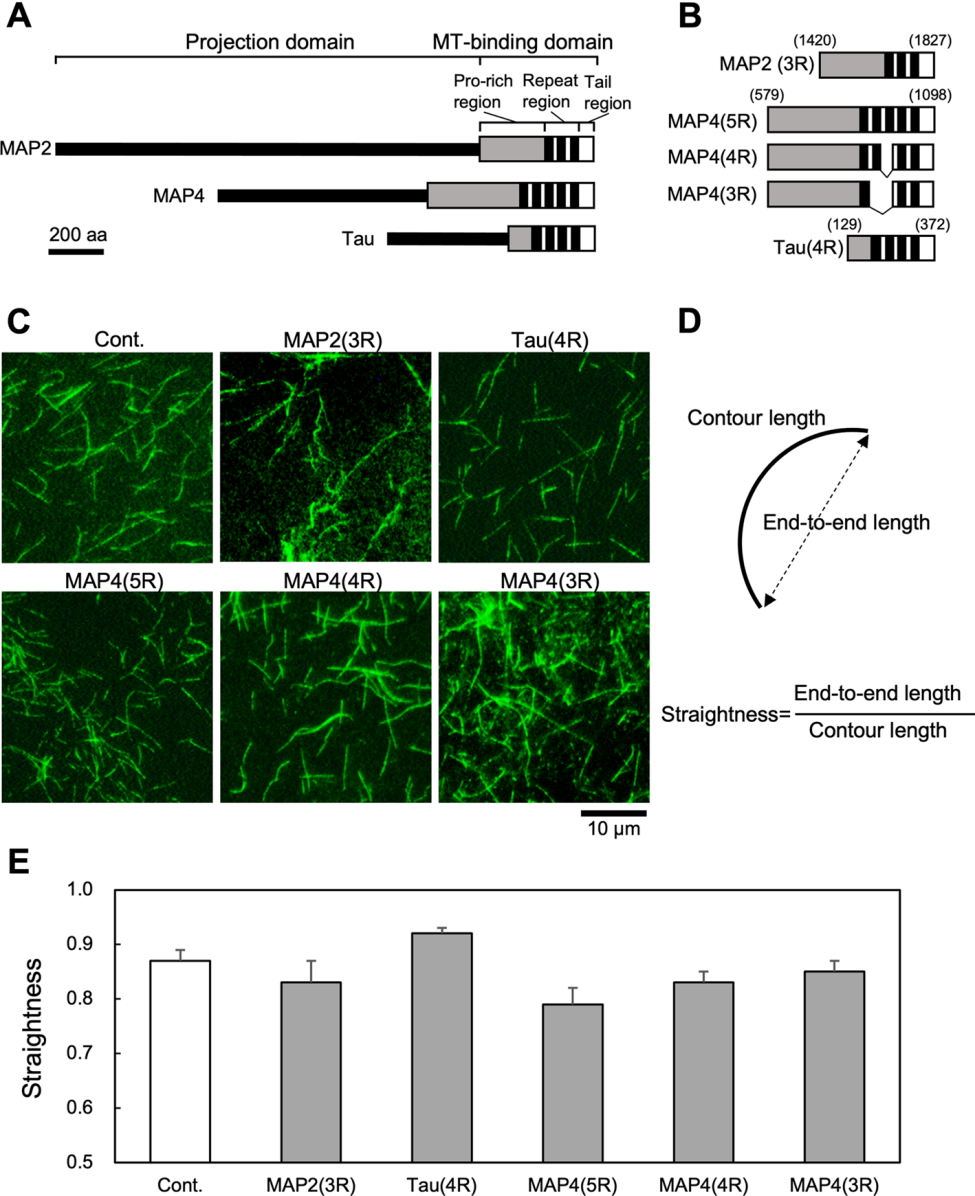
Schematic illustration of MBD fragments of MAPs used in this study and estimation of rigidities of the MAPs-bound microtubules. (**A**) Schematic structures of MAP2, MAP4, and tau superfamily proteins. The structures are classified into a projection domain and a microtubule-binding domain (MBD). MBDs are further separated into Pro-rich, repeat, and tail regions. The repeat region consists of tandemly repeated AP sequences shown as black boxes. (**B**) Structures of MBD fragments of MAPs used in this study. The numbers indicate amino acid residue number. Three isoforms of MAP4 with different repeat numbers were also used. The number before R in parentheses indicates the number of repeats. (**C**) Fluorescence microscopic observation of microtubules bound to MBD fragments of MAPs. 500 nM tubulin dimers labeled with DyLight488 was mixed with 20 nM of MBD fragments of MAPs in the presence of 15 µM taxol, and incubated for 60 min at 37 °C. Samples were observed by fluorescence microscopy using a 100 × objective lens. (**D**) Definition of straightness. The straightness of microtubules was defined as end-to-end length/contour length. If microtubules are straight, then straightness = 1. (**E**) Straightness of microtubules (n = 50) without (Cont.) or with MAP fragments.

To investigate whether MAP2, MAP4, and tau (Fig. 1A) interact with microtubules to affect their physical properties, we used MBD fragments (Fig. 1B) of the MAPs in this study. There are isoforms with a different number of repeat sequences in these MAPs^9^. To investigate whether different numbers of repeats affect the physical properties of microtubules differently, we also evaluated three MAP4 isoforms with different repeat numbers. These MBD fragments were prepared using recombinant *E. coli* as was previously reported^19,31^. The result of SDS-PAGE of the MBD fragments are shown in Fig. S1.

**Figure 2:**
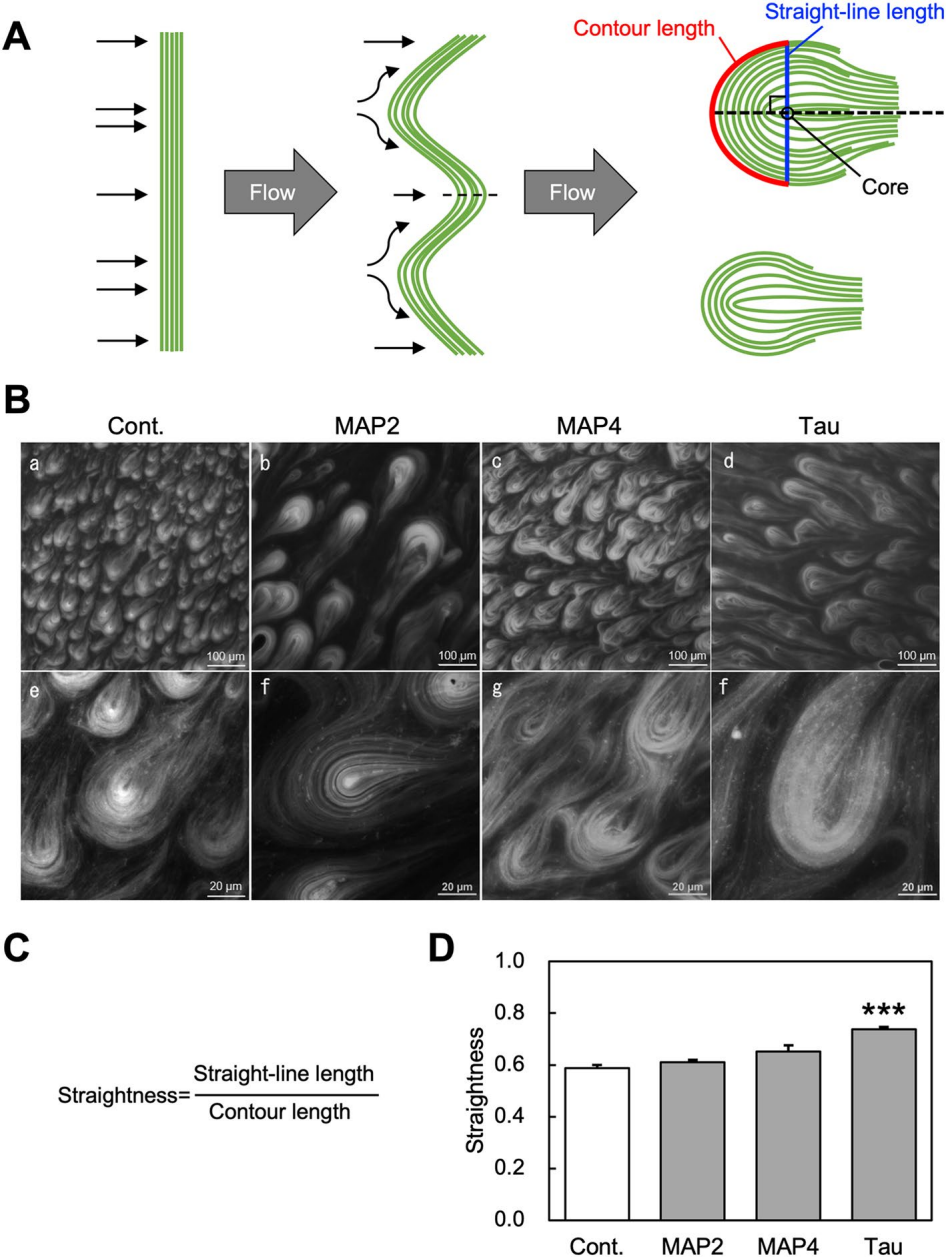
Evaluation of the effect of MAPs on the flexural rigidity of microtubules by analyzing their teardrop pattern. (**A**) A schematic model of teardrop pattern formation. The straightness of the head region of teardrop patterns was measured from randomly selected independent teardrops. Blue and red lines indicate straight-line length through teardrop core and contour length, respectively (top right teardrop). (**B**) Fluorescence microscopic images of teardrop patterns formed in the presence or absence (Cont.) of microtubule-binding domain (MBD) fragments of MAPs. 135 µM of taxol-stabilized microtubules labeled with DyLight488 was mixed with 13.5 µM of MBD fragments of MAPs, forming teardrop patterns. MAP2, MAP4, and tau are MAP2(3R), MAP4(5R), and tau(4R), respectively in Fig. 1B. The upper row (a–d) and lower row (e–f) are low-magnification and high-magnification microscopic images, respectively. (**C**) Definition of straightness. In this case, the straightness of microtubules was defined as straight-line length/contour length. (**D**) Relationship between types of MAPs added and straightness (n = 10). *** denote *P* < 0.001 for Cont., as determined by a Mann–Whitney U test.

MBD fragments of MAP4 induced microtubule bundles, although bundling activity differed depending on the number of repeat sequences^19^. Since the bundling of microtubules affects the flexural rigidity of microtubules, in this study, we observed the morphology of microtubules in the presence of substoichiometric amounts of MAPs in which no bundles had formed. It was reported that even low concentrations of MAP2 and tau promoted a substantial increase in microtubule rigidity^30^. Therefore, we predicted that even in the presence of substoichiometric amounts of MAPs, their effect on the flexural rigidity of microtubules could be quantified. When observing taxol-stabilized microtubules in the presence of MBD fragments of MAPs under a fluorescence microscope (Fig. 1C), we noticed a slight difference in the shape of microtubules. In particular, the tau-bound microtubules appeared to have a straight and needle-like form. Therefore, we estimated the straightness of MAP-bound microtubules from the contour length of the filament and the end-to-end length of filaments (Fig. 1D). The result showed that the straightness of tau-bound microtubules was greater than control microtubules without MAPs (Fig. 1E). It was also shown that the straightness of microtubules bound to MAP2 and the MAP4 isoform containing five repeats was smaller than that of control microtubules (Fig. 1E). Microtubule straightness was similar among MAP4 isoforms with a different number of repeats (MAP4(5R), MAP4(4R), and MAP4(3R)) (Fig. 1E). These results suggest that tau-bound microtubules are straighter and less bendable than MAP2- or MAP4-bound microtubules. However, since these observations were performed under the condition in which microtubules were free and unloaded, the flexural rigidity of microtubules might not have been evaluated correctly. Therefore, we next observed the bending of MAP-bound microtubules under load-applied conditions by hydrodynamic-flow (Fig. 2).

### Evaluation of the flexural rigidity of MAP-bound microtubules by analyzing the teardrop pattern

Hydrodynamic-flow induces a teardrop pattern that is formed from a bunched microtubule^34^. Since multiple microtubules are bunched in neurites, which we focus on in this study, we evaluated the flexural rigidity of the bunched microtubules using teardrop patterns (Fig. 2A). The teardrop patterns of taxol-stabilized microtubules which were formed in the presence (Fig. 2B, MAP2, MAP4, and tau) or absence (Fig. 2B, Cont. and Movie S1) of MAP fragments, were observed by fluorescence microscopy. Analyses of the shape of the teardrops’ patterns indicated that the MAP4- and tau-bound microtubules were straighter than those of the control (Fig. 2D). This result was consistent with our previous results in which the MBD fragment of MAP4 reduced the flexibility of taxol-stabilized microtubules and made them more prone to breakage by pipetting^18^. Furthermore, this result revealed that straightness of tau-bound microtubules was largest among the tested MAPs. To eliminate the effect of taxol on the flexural rigidity of microtubules, as was previously reported^28^, we also performed the same experiment in the absence of taxol (Fig. S2). The results were comparable to those in the presence of taxol. The straightness of control microtubules without MAP increased by removing taxol, i.e. flexural rigidity increased, but was modest compared to previously reported results^28,30^. This could be an effect of microtubule bundles.

**Figure 3:**
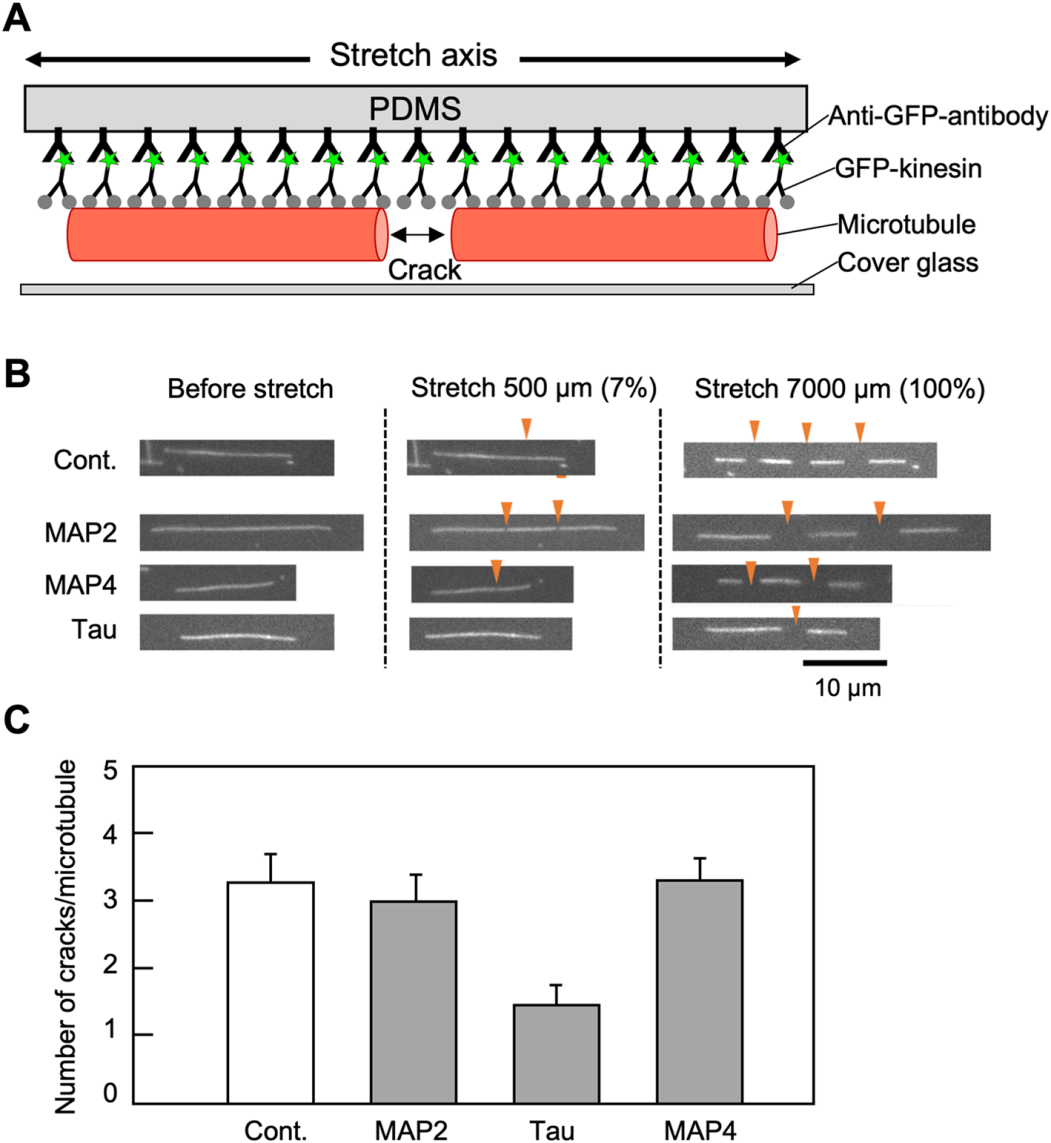
Measurement of tensile strength of MAPs-bound microtubules using a mechanical chamber. (**A**) Schematic diagram of the mechanical chamber. Microtubules composed of tubulin dimers equivalent to 200 nM were immobilized on PDMS bound to the anti-GFP antibody via GFP-fused kinesin, and then PDMS were stretched after the addition of 10 nM MBD fragments of MAPs. MAP2, MAP4, and tau are MAP2(3R), MAP4(5R), and tau(4R), respectively in Fig. 1B. (**B**) Typical fluorescence microscopic images of MAPs-bound microtubules before stretching (left), after stretching 500 µm (middle), and after stretching 7000 µm (right). Orange arrowheads indicate cracks caused by stretching. (**C**) Number of cracks per microtubule after stretching 7000 µm (n = 10).

We also evaluated whether the binding of MAPs affects the tensile strength of microtubules using a mechanical chamber (Fig. 3A), according to our previous reports^35,36^. The results showed that tau-bound microtubules were the least cracked (Fig. 3B and C).

In the in vitro experiments (Figs. 1, 2 and 3), substoichiometric amounts (4–10%) of MDB fragments were added to the microtubules. These results suggest that one molecule of tau allosterically affected the physical properties of multiple tubulin protomers, as was robustly established for interactions between actin filaments and actin-binding proteins^37^.

### Analysis of the effect of MAPs on the morphology of microtubules in cells

**Figure 4:**
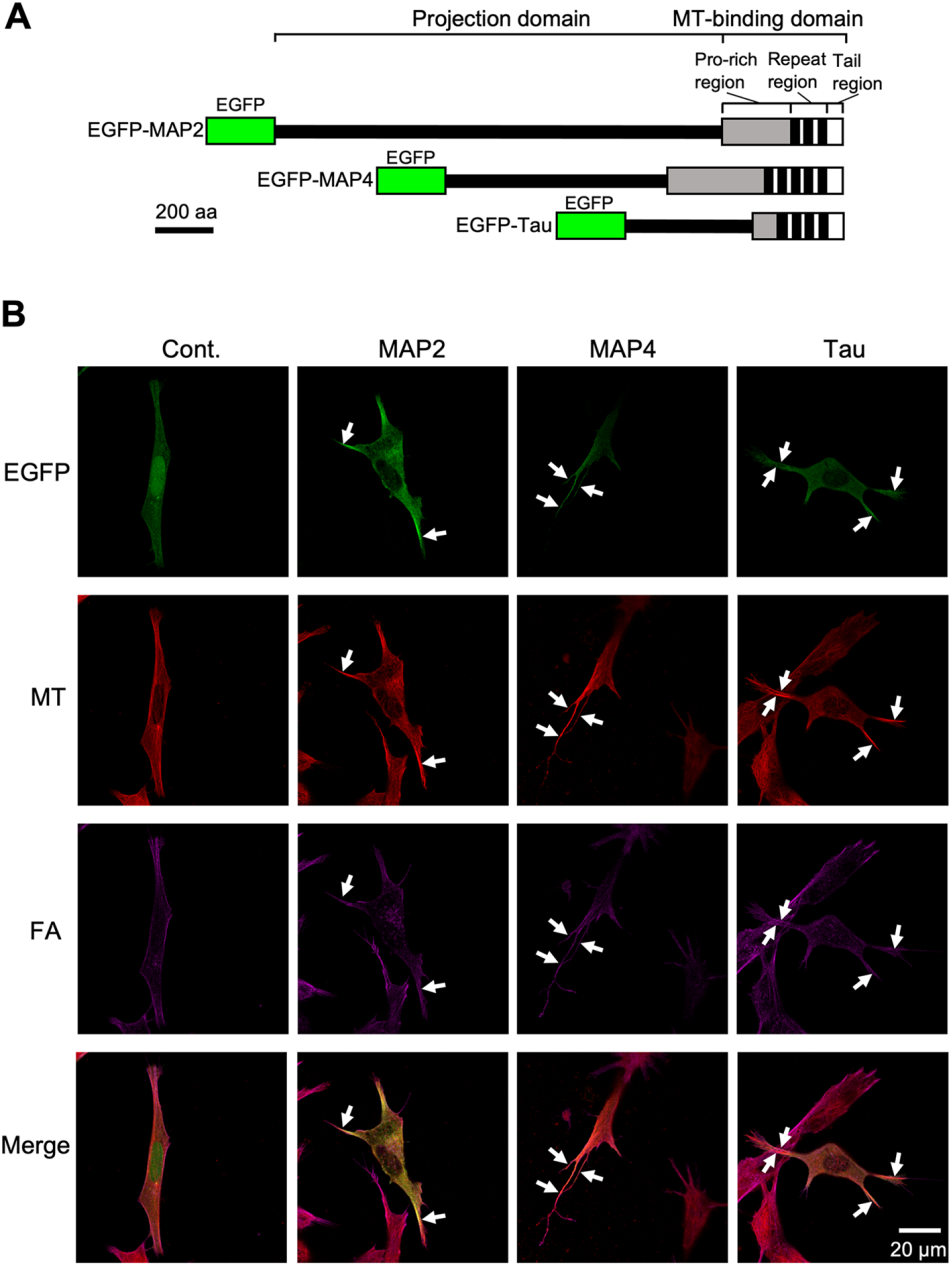
Confocal microscopic images of paraformaldehyde-fixed SH-SY5Y cells expressing EGFP-MAPs. (**A**) Schematic structures of EGFP-MAPs. EGFP was fused to the N-terminal region of each full-length MAP. (**B**) SH-SY5Y cells were transfected with EGFP (Cont.) or EGFP-MAPs (MAP2, MAP, and tau) constructs, fixed with paraformaldehyde, then observed by confocal microscopy using a 100 × objective lens. From top to bottom, EGFP, microtubules (MT), actin filaments (FA), and their merged images are shown. White arrows indicate protrusions that enclose thick microtubule bundles.

Several papers reported that the type of MAP has different effects on the formation of cell protrusions^38–40^, but the effect on the morphology of individual microtubules in these protrusions is not well understood. Therefore, we used a confocal laser scanning microscope to analyze in detail the morphology of microtubules in the protrusions of human neuroblastoma SH-SY5Y cells expressing each EGFP-fused full-length MAP (Fig. 4A).

SH-SY5Y cells were separately transfected with EGFP, EGFP-MAP2, EGFP-MAP4, and EGFP-tau expression plasmids, and observed by confocal microscopy after fixation with paraformaldehyde (Fig. 4B). Microscopic observations revealed that cells expressing EGFP-MAPs had more protrusions containing thick microtubule bundles than the control (Fig. 4B, white arrows). The proportions of cells with protrusions containing thick microtubule bundles per EGFP-expressing cell were as follows: control (28%, n = 32), MAP2 (64%, n = 36), MAP4 (65%, n = 68), and tau (86%, n = 50). However, individual microtubules bound to EGFP-MAPs could not be clearly observed in cells fixed with paraformaldehyde, so fixation was then performed with acetone (Fig. S3). Confocal microscopic observations clearly showed the co-localization of microtubules and EGFP-MAPs, demonstrating that EGFP-MAPs can bind to microtubules in cells (Fig. S3).

**Figure 5:**
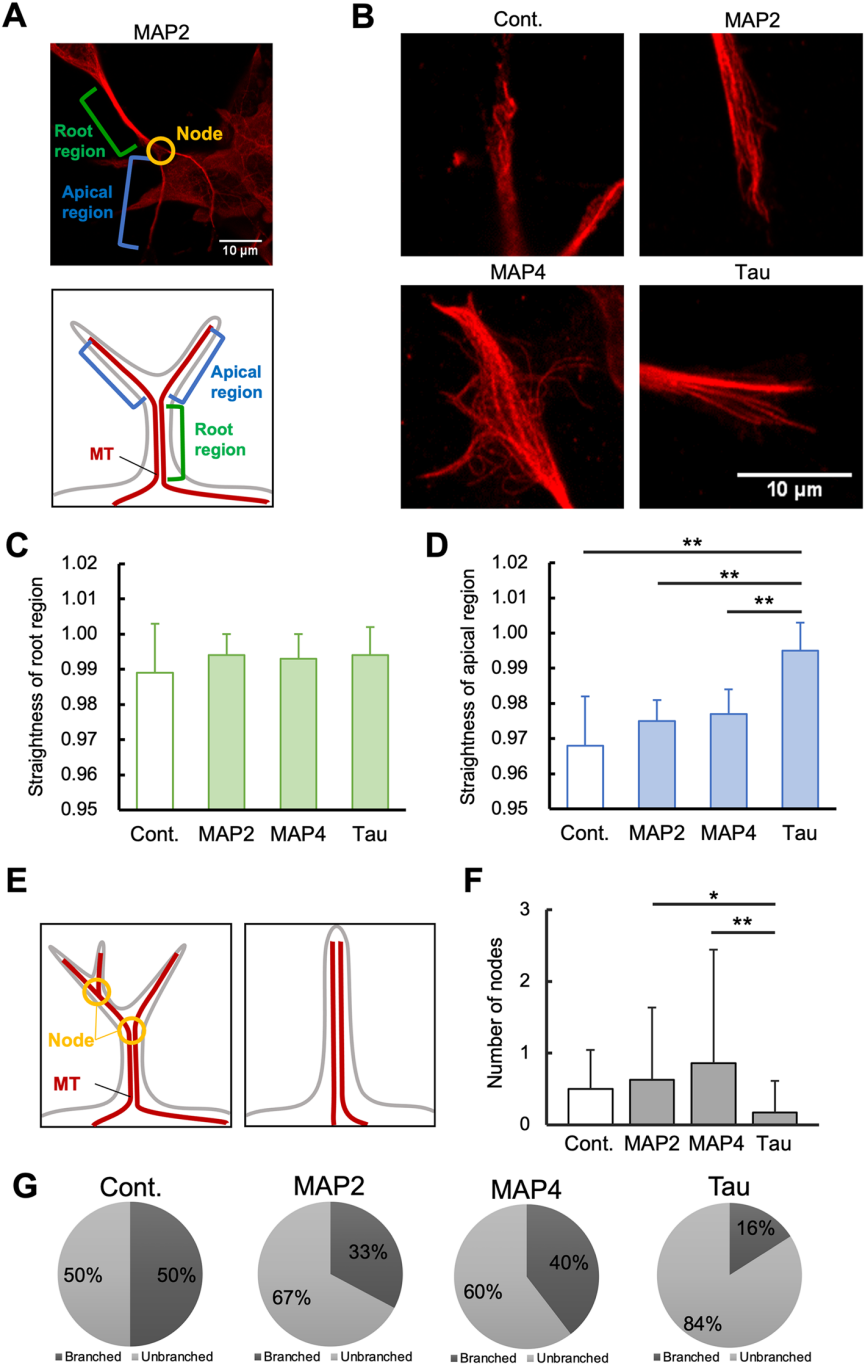
Microtubule bundles present in the protrusions of SH-SY5Y cells expressing EGFP-tau have straighter microtubules in the apical region and fewer branches than cells expressing EGFP-MAP2 and EGFP-MAP4. (**A**) An image of a cell protrusion expressing EGFP-MAP2. In this study, the tip of the branch (node) is defined as the apical region, and the part from the cell body to the first branch is defined as the root region. (**B**) Images of typical apical regions of a cell protrusion expressing EGFP or EGFP-MAPs (MAP2, MAP4, and tau). (**C**, **D**) Rigidities of the root region (**C**) and apical region (**D**) of a cell protrusion expressing each EGFP-MAP. Rigidities were calculated as: straight line length at the edges of region/length along the region of protrusions containing microtubule bundles. (**E**) Quantification of cell branching. The schematic diagrams show that the number of nodes is 2 (left) or 0 (right). (**F**) Node number of cell protrusions expressing each EGFP-MAP. (**G**) The ratio of branched protrusion and unbranched protrusion per cell. Number of cells used for evaluation: EGFP (n = 6), EGFP-MAP2 (n = 12), EGFP-MAP4 (n = 24), EGFP-tau (n = 24). * and ** denote 0.01 < *P* < 0.05 and 0.001 < *P* < 0.01, respectively, as determined by a Mann–Whitney U test.

Next, we observed in detail the morphology of microtubules in cell protrusions expressing EGFP-MAPs (Fig. 5). Since branching was observed in the protrusions of cells expressing MAPs, the microtubules of the protrusions were analyzed separately in the root and apical regions (Fig. 5A). Typical images of the apical regions (Fig. 5B) showed that microtubules in protrusions expressing EGFP-MAP2 and EGFP-MAP4 were wavy, similar to control cells expressing EGFP alone. In contrast, the microtubules in the protrusions expressing EGFP-tau were straight, like needles. The rigidities of microtubules in the protrusions revealed that the three MAPs tended to induce straight microtubules in the root region, although there were no significant differences between them (Fig. 5C). The three MAPs also induced straight microtubules in the apical region, but EGFP-tau expression induced significantly straighter microtubules than EGFP-MAP2 and EGFP-MAP4 (Fig. 5D). We also quantified the number of nodes in order to quantify the number of branches (Fig. 5E, left). The results show that the number of nodes in EGFP-tau-expressing cells was significantly smaller than cells expressing EGFP-MAP2 and EGFP-MAP4 (Fig. 5F). These results suggest that tau induced the formation of straight and unbranched protrusions (Fig. 5E, right). When the ratio of branched protrusions and unbranched protrusions per cell was measured, EGFP-tau-expressing cells had the highest ratio of unbranched protrusions (Fig. 5G). To confirm whether the effect of MAPs on cell protrusion depends on their expression level, we semi-quantified the expression level of EGFP-MAPs from microscopic images (Fig. S4). The result suggests that only the expression level of EGFP-MAP2 was significantly lower, probably due to the molecular size of MAP2, which was largest among all EGFP-MAPs. On the other hand, there was no significant difference in the expression levels of EGFP (control), EGFP-MAP4, and EGFP-tau. These results demonstrate that the characteristics of EGFP-tau-expressing cells are independent of their expression levels.

## Discussion

The microtubule cytoskeleton is the backbone of cells, so its mechanical properties greatly affect their shape and properties. In this study, we demonstrated that the microtubule-binding domains of the MAP2/MAP4/tau superfamily had different effects on the mechanical properties of microtubules and may also affect the morphogenesis of cell protrusion. In vitro experiments quantitatively demonstrated that the binding of MAPs to microtubules increased stiffness (Fig. 2), as was previously reported^18,28,30^. Interestingly, the effect of tau was significantly higher than that of MAP2 and MAP4 (Figs. 2, S2). In this study, substoichiometric amounts (4–10%) of MBD fragments were added to the microtubules during in vitro experiments (Figs. 1, 2 and 3). Calculation of the microtubule-binding ratio under each experimental condition from the concentrations of tau fragments and *K*_*d*_ value indicated that the tau fragments bound to microtubules at a molar ratio of 3.41–9.99% (Table S3). This suggests that tau allosterically affected the physical properties of multiple tubulin protomers.

We also expressed these MAPs in SH-SY5 cells and analyzed the shape of individual microtubules in cell protrusions in detail using confocal microscopy. The results show that the microtubules in the apical region of tau-expressing cell protrusions had straighter microtubules than cells expressing MAP2 and MAP4 (Fig. 5D). Furthermore, the branching of protrusions of tau-expressing cells was less than that of MAP2- and MAP4-expressing cells (Fig. 5F and G). These results suggest that the properties of the microtubule cytoskeleton regulated by the binding of MAPs contribute to the morphogenesis of neurite tips. In neuronal function of the mammalian brain, the neuron transmits information via an axon and receives input via a dendrite^41^. MAP2, MAP4, and tau stabilize microtubules and may help the formation of dendrites and axons, allowing the properties of microtubules to be characterized. MAP2 and MAP4 localize to the dendrite with branching and flexible protrusions whereas and tau localizes to the axon with a long, straight, and constant diameter^27,42^. The morphology of dendrites receiving input and axons with access to other more distant neurons may contribute to efficient neuronal communication^41^. We speculate that these MAPs provide a morphological advantage, having a characteristic composition of microtubules in the dendrite and axon.

Hawkins et al. reported that the order in which tau was added had different effects on microtubule rigidity^43^. Microtubule rigidity increased dramatically when tau was copolymerized with tubulin and then stabilized with taxol, but not when tau was added after stabilizing microtubules with taxol. In the former case, tau may bind to the microtubule lumen rather than to the outside^44^. Since the focus of our study was on how differences in MAPs bound to formed microtubules affect, MAPs were added to stabilized microtubules in taxol during in vitro experiments (Figs. 1, 2 and 3). However, *in cellulo* experiments (Figs. 4, 5) included both microtubules formed by copolymerization of MAPs and tubulin, as well as MAPs subsequently bound to formed microtubules. Therefore, a detailed evaluation of the flexural rigidity of MAPs-microtubule complexes formed under the same conditions as in cells will be the focus of future work.

Although some reports indicated that tau induced long neurites^40,45,46^, those reports focused on the bundling of microtubules rather than the rigidity of individual microtubules and discussed the mechanism of neurite formation. In this study, we also showed that MAP2, MAP4, and tau all induced protrusions containing thick microtube bundles (Fig. 4B, white arrows), and that the rigidity of the root region did not differ between MAPs (Fig. 5C). On the other hand, our results revealed that a single tau-bound microtubule was straighter and harder to bend than a single MAP2- or MAP4-bound microtubule in vitro (Fig. 1). These results suggest that a role of MAPs, which define the morphology and properties of cell protrusions, is to control the flexural rigidity of individual microtubules, in addition to traditional microtubule bundle formation. The MAP2/MAP4/tau superfamily has many phosphorylation sites, and by phosphorylating them with CDK1^10^, PKC^11^, GSK^12^, and MARK^13^, etc., the affinity for microtubules is finely adjusted. These results suggest that the effect of MAPs on the flexural rigidity of microtubules can also be regulated by their phosphorylation.

The microtubule-binding domains of the MAP2/MAP4/tau superfamily have similar primary structures^9,47^. In particular, the repeat region sequence is highly conserved. To discover clues related to the region in the primary structures that interact with microtubules to make them straighter microtubules, we examined the molecular phylogenetics of each domain and subdomain of these MAPs (Fig. S5). The results confirmed that in most phylogenetic trees (Fig. S5, full length, MBD, Pro-rich region, tail region), non-neuronal MAP4 is phylogenetically distant from neural MAP2 and tau. On the other hand, it was revealed that tau was phylogenetically distant from MAP2 and MAP4 only in the phylogenetic tree of the repeat region consisting of tandem repeat sequences and inter repeat sequences (Fig. S5, repeat region). Therefore, we only focused on tau amino acid sequences of the repeat region that differed from those of MAP2 and MAP4 (Fig. S6). The comparison of amino acid sequences revealed four amino acids in tau whose side-chain properties differed significantly from those of MAP2 and MAP4 (Fig. S6 #1–4). Mutations in these amino acids in the process of molecular evolution of MAPs may have given tau the ability to straighten microtubules. We previously reported that MAP4 may have acquired the second repeat sequence (R2) in the course of its evolution^48^. The flexural rigidities of MAP4- and MAP2-bound microtubules were similar, suggesting that the amino acids in the sequence between R3 and R5 (Fig. S6), but not around R2, affect the flexural rigidity of microtubules.

An early Cryo-EM study revealed that MAP2 and tau bind longitudinally along the outer ridges of microtubule protofilaments^49^. A higher-resolution structural analysis was then performed using Cryo-EM to elucidate the structure of the binding region between one of the tandemly repeated repeats in tau^50^ and MAP4^24^ MBDs and microtubules. However, members of the MAP2/MAP4/tau superfamily are intrinsically disordered proteins, so it is difficult to elucidate how each repeat sequence and its inter-repeat sequences interact with microtubules because of the low density due to its flexibility^24^. Among the four amino acid residues focused on in this study (Fig. S6), #1, #2, and #3 displayed significantly different charges between tau and MAP2/MAP4. There are many reports on the interaction between acidic residues of tubulin and basic amino acid residues of MAPs^50,51^. Therefore, it is possible that flexural rigidity is controlled by the interaction of the amino acid residues #1, #2, or #3 with microtubules. The amino acids that affect the mechanical properties of microtubules may need further investigation from the viewpoint of structural biology.

## Methods

### Materials

Paclitaxel and Alexa Fluor 647 phalloidin were purchased from AdipoGen (San Diego, CA, USA) and Life Technologies (San Diego, CA, USA), respectively. HiLyte Fluor™ 488-labeled tubulin was purchased from Cytoskeleton Inc. (Denver, CO, USA). A monoclonal anti-β-tubulin antibody (T4026) and a secondary antibody with Alexa Fluor 546 (A11003) were purchased from Sigma-Aldrich (St. Louis, MI, USA) and Thermo Fisher Scientific (Waltham, MA, USA), respectively. Restriction enzymes, PrimeSTAR Max DNA Polymerase, Ligation Mix, and In-Fusion HD Enzyme Premix were purchased from Takara Bio (Tokyo, Japan) and Rosetta (DE3) pLys was purchased from Novagen (Darmstadt, Germany). Human neuroblastoma, SH-SY5Y cells (EC94030304-F0) were purchased from KAC (Kyoto, Japan). All other chemicals were purchased from Wako Pure Chemical Industries (Osaka, Japan).

### Protein preparation

Porcine brain tubulin was prepared by a standard method^5,52^. Porcine brain was purchased from the HOKUREN Federation of Agricultural Cooperatives (Hokkaido, Japan). MBD fragments of MAPs were expressed in *Escherichia coli* (Rosetta (DE3) pLys) and purified according to our previous reports^19,23,31^. Briefly, the heat-stable fraction of each extract was subjected to successive column chromatography using a phosphocellulose UNOsphere™ S column (Bio-Rad Laboratories Inc., Hercules, CA, USA) and a TOYOPEARL® Butyl-650 column (Tosoh Co., Ltd., Tokyo, Japan). In UNOsphere™ S and TOYOPEARL® Butyl-650 column chromatography, bound MAPs were eluted with gradients of 0 to 1 M NaCl and 1.2 M to 0 M (NH_4_)_2_SO_4_, respectively. Eluted MAPs fragments were dialyzed against 20 mM MES (pH 6.8), 0.5 mM MgCl_2_, and 0.1 mM EGTA containing 0.5 mM para-methyl-sulphonyl-fluoride. The purity of the purified proteins was confirmed by SDS-PAGE^53^ and their concentration was assessed by Lowry’s method^54^.

### Evaluation of rigidity of MAPs-bound microtubules

Fluorescently-labeled microtubules were prepared by copolymerization of HiLyte Fluor™ 488-labeled tubulin and purified wild-type tubulin at a 1:14 molar ratio. To induce polymerization, 10 µM of tubulin dimers mixture was incubated in 100 mM MES (pH 6.8), 0.5 mM MgCl_2_, 0.1 mM EGTA, 1 mM GTP, and 15 µM paclitaxel for 1 h at 37 °C. Five hundred nM of microtubules labeled with DyLight488 was mixed with 20 nM MBD-fragments of MAPs, and the mixtures were incubated for another 60 min at 37 °C. The samples, which were sandwiched between a glass slide and a coverslip, were observed at 25 °C under a fluorescence microscope (TE2000, Nikon, Tokyo, Japan) equipped with a color CCD camera (DP72, Olympus, Tokyo, Japan) and a 100 × objective lens (Plan Apo λ 100 × /1.45 Oil, Nikon). To estimate the straightness of microtubules, the linear distance between microtubule ends and contour length was measured using ImageJ software (NIH, Bethesda, MD, USA).

### Evaluation of flexural rigidity of MAPs-bound microtubules by measuring teardrop patterns

Fluorescently-labeled microtubules were prepared by copolymerization of HiLyte Fluor™ 488-labeled tubulin and purified wild-type tubulin at a ratio of 1:50. 135 µM of tubulin dimers mixture was incubated in 100 mM MES (pH 6.8), 0.5 mM MgCl_2_, 0.1 mM EGTA, and 1 mM GTP in the presence or absence of paclitaxel for 1 h at 37 °C to induce microtubules, then mixed with MBD fragments of MAPs and incubated for another 30 min at 37 °C. Teardrop patterns of the MAPs-bound microtubules were formed using an established method^34^, and observed at 25 °C under a fluorescence microscope (ECLIPSE Ni, Nikon) equipped with a CMOS camera (DS-Qi2, Nikon) and an 100 × objective lens (Plan Apo λ 100 × /1.45 Oil, Nikon). The flexural rigidity of microtubules forming teardrops was estimated from the straightness of the head region of teardrop patterns. In this case, the straightness of microtubules was defined as the straight-line length through a teardrop core/contour length.

### Evaluation of tensile strength of MAPs-bound microtubules using a mechanical chamber

The tensile strength of MAPs-bound microtubules was evaluated using a mechanical chamber according to a previous report^55^. Briefly, rhodamine-labeled microtubules composed of tubulin dimers equivalent to 200 nM were immobilized on elastic medium, polydimethylsiloxane (PDMS) was bound to the anti-GFP antibody via GFP-fused kinesin, and then PDMS was stretched after the addition of 10 nM of MBD fragments of MAP2, MAP4, and tau.

### Construction of expression plasmids for EGFP-fused human MAPs

Human full-length MAP4 and MAP2 cDNAs were purchased from Danaform (MAP4 clone ID: H013078A70, MAP2 clone ID: 100068957). Human full-length tau cDNA was purchased from Addgene (VN-Tau (wt) catalog no.: 87368). The sequences of the purchased cDNAs correspond to MAP4 (accession no.: NM_002375), MAP2 (accession no.: NM_002374.3), and tau (accession no.: NM_005910.5) of the National Center for Biotechnology Information (NCBI). The DNA fragments of each MAP were amplified by the polymerase chain reaction (PCR) using PrimeSTAR® Max DNA Polymerase (Takara Bio). All PCR primer sets, which are shown in Supplementary Table 1, were purchased from Hokkaido System Science (Sapporo, Japan). PCR of full-length MAP4 and MAP2 was performed in 30 cycles of denaturation at 98 °C for 10 s, annealing at 55 °C for 5 s, and extension at 72 °C for 30 s. PCR of full-length tau was performed in 35 cycles of denaturation at 98 °C for 10 s, annealing at 55 °C for 5 s, and extension at 72 °C for 8 s. In a previous study, we constructed pEGFP-C3-MAP4-LP with DNA expressing mouse full-length MAP4 at the *Bg*lII/*Hind*III cleavage site of pEGFP-C3 (Clontech, Tokyo, Japan)^31^. Using this pEGFP-C3-MAP4-LP as a template, linearization vector EGFP-C3 was obtained by inverse PCR using PrimeSTAR® Max DNA Polymerase (Takara Bio) and the EGFP-C3 primer set defined in Supplementary Table 1. PCR of EGFP-C3 was performed in 30 cycles of denaturation at 98 °C for 10 s, annealing at 55 °C for 5 s, and extension at 72 °C for 30 s. The PCR product was gel-purified using NucleoTrap® (MACHEREY–NAGEL, Düren, Germany). In-Fusion HD Enzyme Premix (Takara Bio) was used to bind full-length MAP4 and EGFP-C3 at the homologous terminal 15 bases. The reaction solution was transformed into *E. coli* DH5α-competent cells (Takara Bio), and the cultured cells were purified using the QIAprep® Spin Miniprep Kit (QIAGEN, Hilden, Germany) to purify the pEGFP-MAP4 (human) plasmid. pEGFP-MAP2 (human) and pEGFP-tau (human) were constructed in a similar manner. In addition, the 5′-end of the linearization vector EGFP-C3 was phosphorylated with T4 Polynucleotide Kinase (Takara Bio), and then self-ligation was performed using Ligation Mix (Takara Bio) to construct the pEGFP-C3 plasmid.

### Cell culture, transfection, and observation of cells expressing EGFP-MAPs

SH-SY5Y cells were maintained in Dulbecco’s modified Eagle’s medium (Wako Pure Chemical Industries) supplemented with 10% fetal calf serum and 0.001% penicillin/streptomycin at 37 °C in 5% CO_2_. The cells were cultured on a round coverslip (Matsunami Glass, Osaka, Japan) coated with poly-d-lysine (0.1 mg/mL) and transfected with pEGFP-MAP4, pEGFP-MAP2, or pEGFP-tau, which are mammalian expression plasmids encoding full-length MAP proteins with its C-terminal fused to EGFP. In this transfection, we used SuperFect transfection reagent (QIAGEN) according to the manufacturer’s instructions. The cells were cultured for 1 day, after which the coverslips were treated with 4% paraformaldehyde phosphate buffer solution (Wako Pure Chemical Industries) for 20 min at 20 °C and washed twice in PBS. Cells were then treated with PBS containing 0.2% Triton X-100 for 5 min and washed three times in PBS. When acetone fixation was used, cells were treated with acetone at − 20 °C for 15 min and not permeated with Triton X-100. After blocking with PBS containing 3% BSA (PBSB) for 30 min at 20 °C, the coverslips were incubated in PBSB containing a monoclonal anti-β-tubulin antibody (T4026, Sigma-Aldrich) at a dilution of 1:200 for 1 h and washed three times with PBS for 5 min. The coverslips were incubated in PBSB containing a secondary antibody conjugated with Alexa Fluor 546 (A-11003, Thermo Fisher Scientific) at a dilution of 1:100 for 1 h. Simultaneously, for fluorescent staining of F-actin, the coverslips were incubated in PBSB containing 0.1 mM Alexa Fluor 647 phalloidin for 1 h, washed three times with PBS for 5 min and rinsed with water. The coverslips were mounted using SlowFade Diamond Antifade mountant (Thermo Fisher Scientific). The cells were observed under a fluorescence microscope equipped with a color CMOS camera (DS-Ri2, Nikon) and confocal microscope system (Nikon Eclipse Ti-C2, Nikon). EGFP intensity (mean of gray values) was measured using ImageJ software to compare the expression levels of EGFP-MAPs.

### Statistical analysis

Quantitative data derived from fluorescence microscopic experiments were expressed as the mean ± standard deviation (SD). In the figures, SD values are represented as error bars. Statistical significance was determined using a Mann–Whitney U test with EZR^56^ (Saitama Medical Center, Jichi Medical University, Saitama, Japan), a graphical user interface for R (The R Foundation for Statistical Computing, Vienna, Austria, version 4.1.1). More precisely, it is a modified version of R commander (version 1.54) designed to add statistical functions frequently used in biostatistics. The levels of statistical significance are indicated in the figure legends. *P* values < 0.05 were assumed to be statistically significant.

## Supplementary Information

Supplementary Information 1.

Supplementary Video 1.

## Data Availability

All data analyzed during this study are included in this published article and its Supplementary Information files. Raw data generated during this study are available from the corresponding author on reasonable request.
