# Supplementary material for: Effects of three microtubule-associated proteins (MAP2, MAP4, and Tau) on microtubules’ physical properties and neurite morphology
Source: Sci Rep. 2023 May 31;13:8870. doi: 10.1038/s41598-023-36073-9 (PMC10232483; doi:10.1038/s41598-023-36073-9)
Supplement: Supplementary file 1 — Supplementary Information 1. [file 41598_2023_36073_MOESM1_ESM.pdf]

Supplementary Information

Effects of Three Microtubule-Associated Proteins (MAP2, MAP4, and Tau) on Microtubules' Physical Properties and Neurite Morphology

Kohei Nishida[1], Kosuke Matsumura[1], Miki Tamura[1], Takuto Nakamichi[1], Keiya Shimamori[1], Masahiro Kuragano[1], Arif Md. Rashedul Kabir[2], Akira Kakugo[3], Susumu Kotani[4], Naoki Nishishita[5], Kiyotaka Tokuraku[1]*

[1]Graduate School of Engineering, Muroran Institute of Technology, Muroran, 050-8585 Japan

[2]Faculty of Science, Hokkaido University, Sapporo, 060-0810 Japan

[3]Department of Physics, Graduate School of Science, Kyoto University, Kyoto 606-8502, Japan

[4]Faculty of Science, Kanagawa University, Kanagawa 221-8686, Japan

[5]Regenerative Medicine and Cell Therapy Laboratories, Kaneka Corporation, Kobe, 650-0047, Japan

* Corresponding author: Kiyotaka Tokuraku Ph. D.

Email: tokuraku@mmm.muroran-it.ac.jp

This PDF file includes:

Table S1 to S3

Figures S1 to S6

Legends for Movies S1

Other supplementary materials for this manuscript include the following:

Movies S1

Table S1. Primer sets used in this study.

Names	Sequence
Full-length MAP4-FW*	5′-TACAAGTACTCAGATATGGCTGACCTCAGTCTTG-3′
Full-length MAP4-RV**	5′-CAGAATTCGAAGCTTTTAGATGCTTGTCTCCTGG-3′
Full-length MAP2-FW*	5′-TACAAGTACTCAGATATGGCAGATGAACGGAAAG-3′
Full-length MAP2-RV**	5′-CAGAATTCGAAGCTTTTACAAGCCCTGCTTAGCG-3′
Full-length tau-FW*	5′-TACAAGTACTCAGATATGGCTGAGCCCCGCC-3′
Full-length tau-RV**	5′-CAGAATTCGAAGCTTTCACAAACCCTGCTTGG-3′
EGFP-C3-FW*	5′-AAGCTTCGAATTCTGCAGTC-3′
EGFP-C3-RV**	5′-ATCTGAGTACTTGTACAGCTCGTC-3′

*Forward primers, ** Reverse primers

Table S2. Information of human MAP isoforms used in molecular phylogenetic analysis (Fig. S5).

Name	Isoform	Accession no.	Domain*
Microtubule-associated protein 2	isoform 1	NP_002365.3	3R
Microtubule-associated protein 2	isoform 2	NP_114033.2	3R
Microtubule-associated protein 2	isoform 4	NP_114035.2	4R
Microtubule-associated protein 2	isoform 5	NP_001034627.1	4R
Microtubule-associated protein 2	isoform 6	NP_001350839.1	3R
Microtubule-associated protein 4	isoform 1	NP_002366.2	5R
Microtubule-associated protein 4	isoform 4	NP_001127836.1	5R
Microtubule-associated protein tau	isoform 2	NP_005901.2	2N4R
Microtubule-associated protein tau	isoform 3	NP_058518.1	0N4R
Microtubule-associated protein tau	isoform 4	NP_058525.1	0N3R
Microtubule-associated protein tau	isoform 5	NP_001116539.1	1N4R
Microtubule-associated protein tau	isoform 7	NP_001190180.1	1N3R
Microtubule-associated protein tau	isoform 8	NP_001190181.1	2N3R

*R and N indicate the number of repeats and the number of inserts in the projection domain, respectively.

Table S3. Microtubule binding rate of MAPs in each experimental condition (Figure 1–3), estimated from the dissociation constant.

		MT-binding rate of MAPs (%)		
	K_d for MT (μM) *	Figure 1	Figure 2	Figure 3
MAP2	0.12	3.21	9.99	3.09
MAP4	0.27	2.57	9.98	2.10
Tau	0.09	3.37	9.99	3.41

*K_d values of MAP fragments are from our previous work (J. Biochem. 2020;168(3):295–303, doi:10.1093/jb/mvaa046).

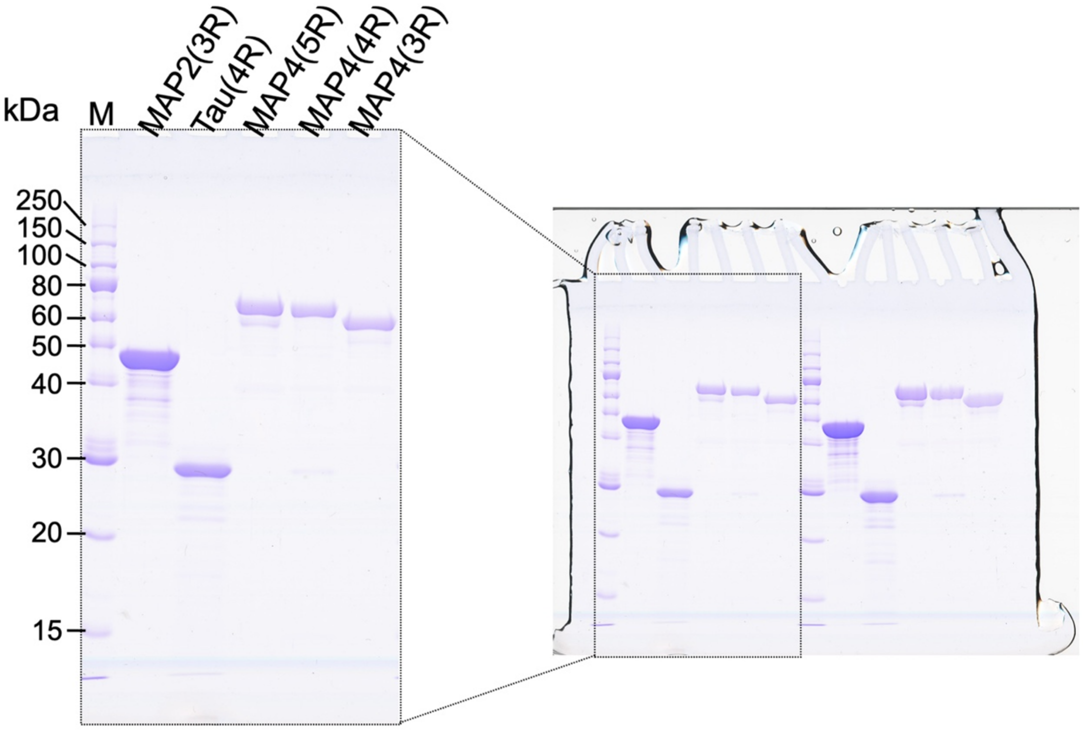

Fig. S1. Electrophoretic patterns of purified MBD fragments. Lane M is the molecular weight marker (CLEARLY Protein Ladder, 3453A, Takara Bio Inc.). The left gel image is a cropped and enlarged image of the boxed section of the right gel image.

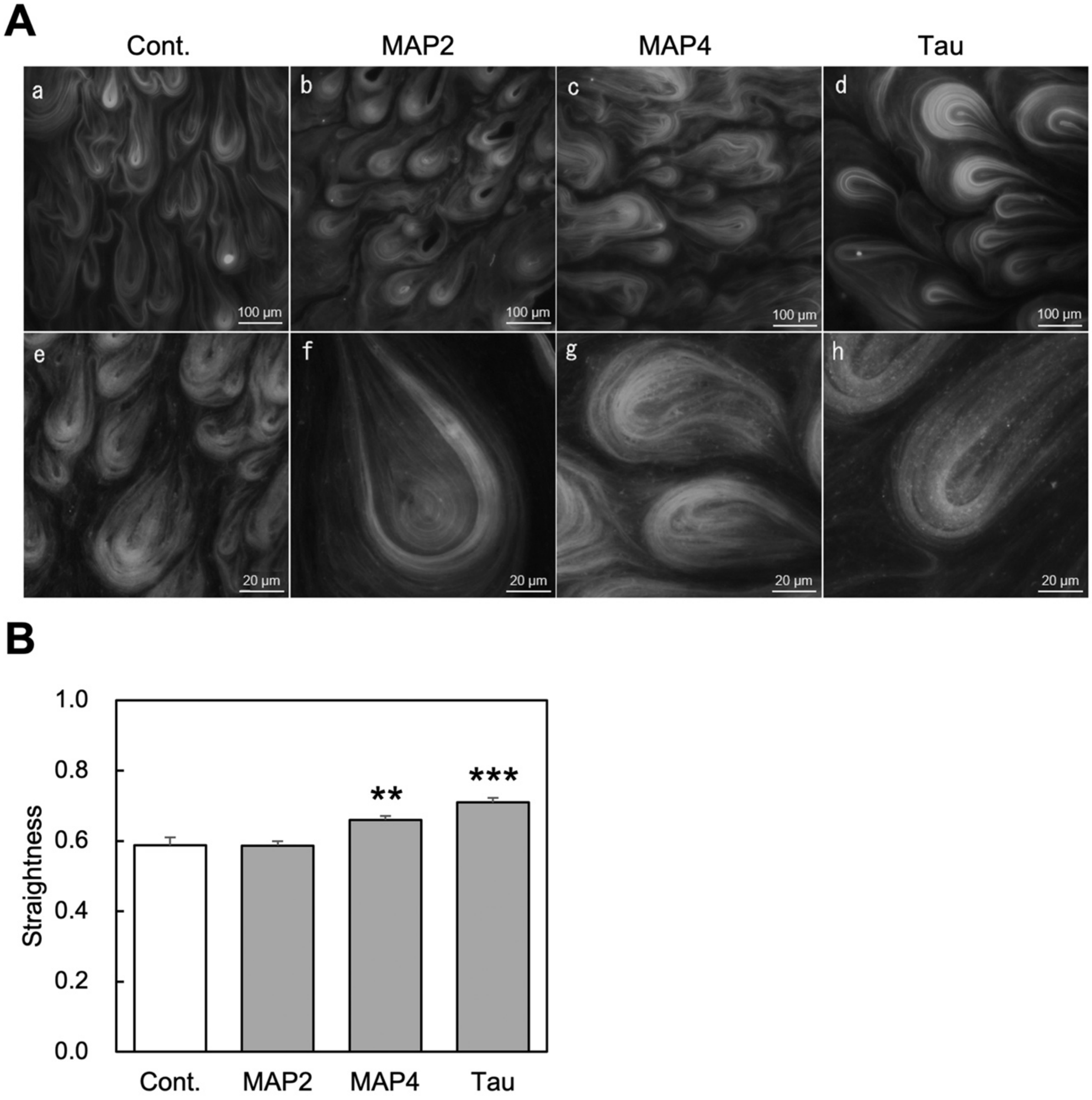

Fig. S2. Evaluation of the effect of microtubule-associated proteins (MAPs) on the flexural rigidity of microtubules by analyzing the teardrop pattern without taxol. (A) Fluorescence microscopic images of teardrop patterns formed in the presence or absence (Cont.) of microtubule-binding domain (MBD) fragments of MAPs. 135 μM of microtubules labeled with DyLight488 were mixed with 13.5 μM of MBD-fragments in the absence of taxol, forming teardrop patterns. The upper row (a-d) and lower row (e-h) are low-magnification and high-magnification microscopic images, respectively. (B) Relationship between types of MAPs added and straightness (n=10). ** and *** denote $0.001 < P < 0.01$ and $P < 0.001$, respectively for the control, as determined by a Mann-Whitney U test.

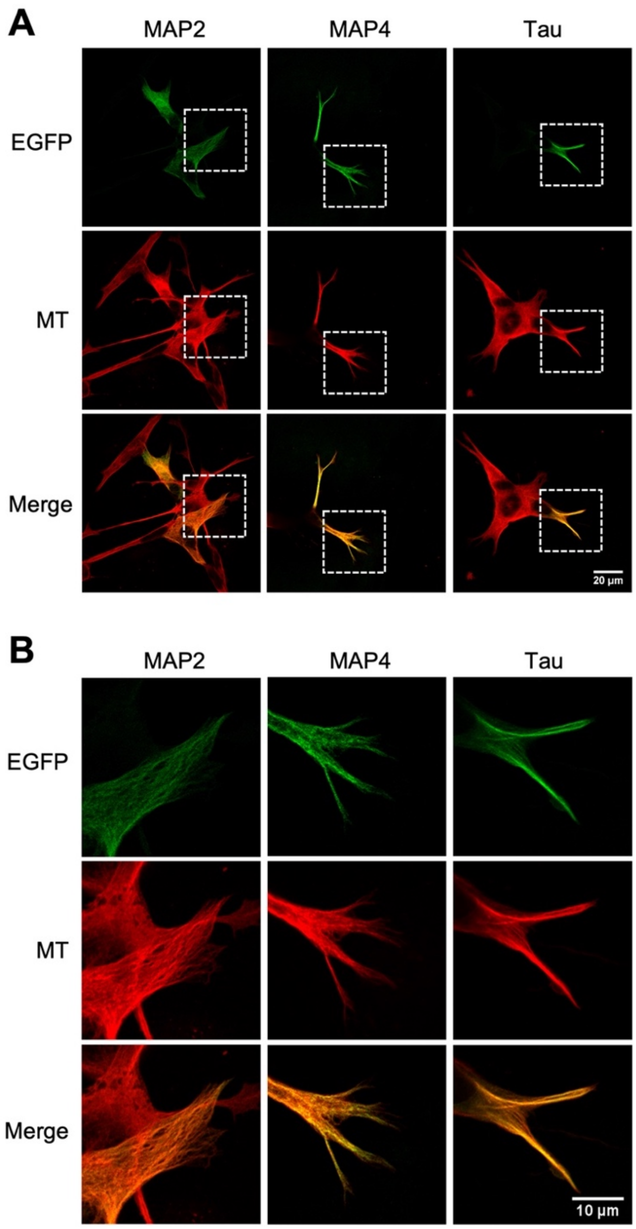

Fig. S3. Confocal microscopic images of acetone-fixed SH-SY5Y cells expressing EGFP-MAPs. (A) SH-SY5Y cells were transfected with EGFP (Cont.) or EGFP-MAPs (MAP2, MAP, and tau) constructs, fixed with acetone, then observed by confocal microscopy using a 100x objective lens. From top to bottom: EGFP, microtubules (MT), and their merged images. (B) Magnified images of the white dashed boxes in (A). Since the EGFP signal could not be detected in EGFP (Cont.)-expressing cells fixed with acetone, fluorescence microscopic images are not shown.

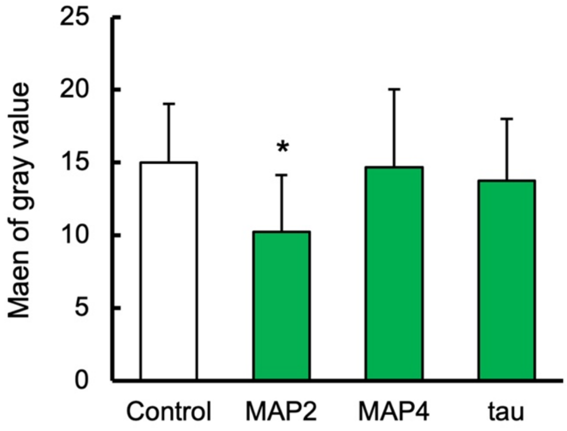

Fig. S4. Expression levels of EGFP-MAPs estimated from the gray value of microscopic images (Fig. 4). SH-SY5Y cells were transfected with EGFP (Cont.) or EGFP-MAP (MAP2, MAP, and tau) constructs, fixed with paraformaldehyde, then observed by confocal microscopy using a 100x objective lens. EGFP intensity of each field was quantified to the mean gray value in the expressed area. The number of fields used for evaluation: Control (n=6), EGFP-MAP2 (n=12), EGFP-MAP4 (n=11), EGFP-tau (n=14). * denotes $0.01 < P < 0.05$ for the Cont. as determined by a Mann-Whitney U test.

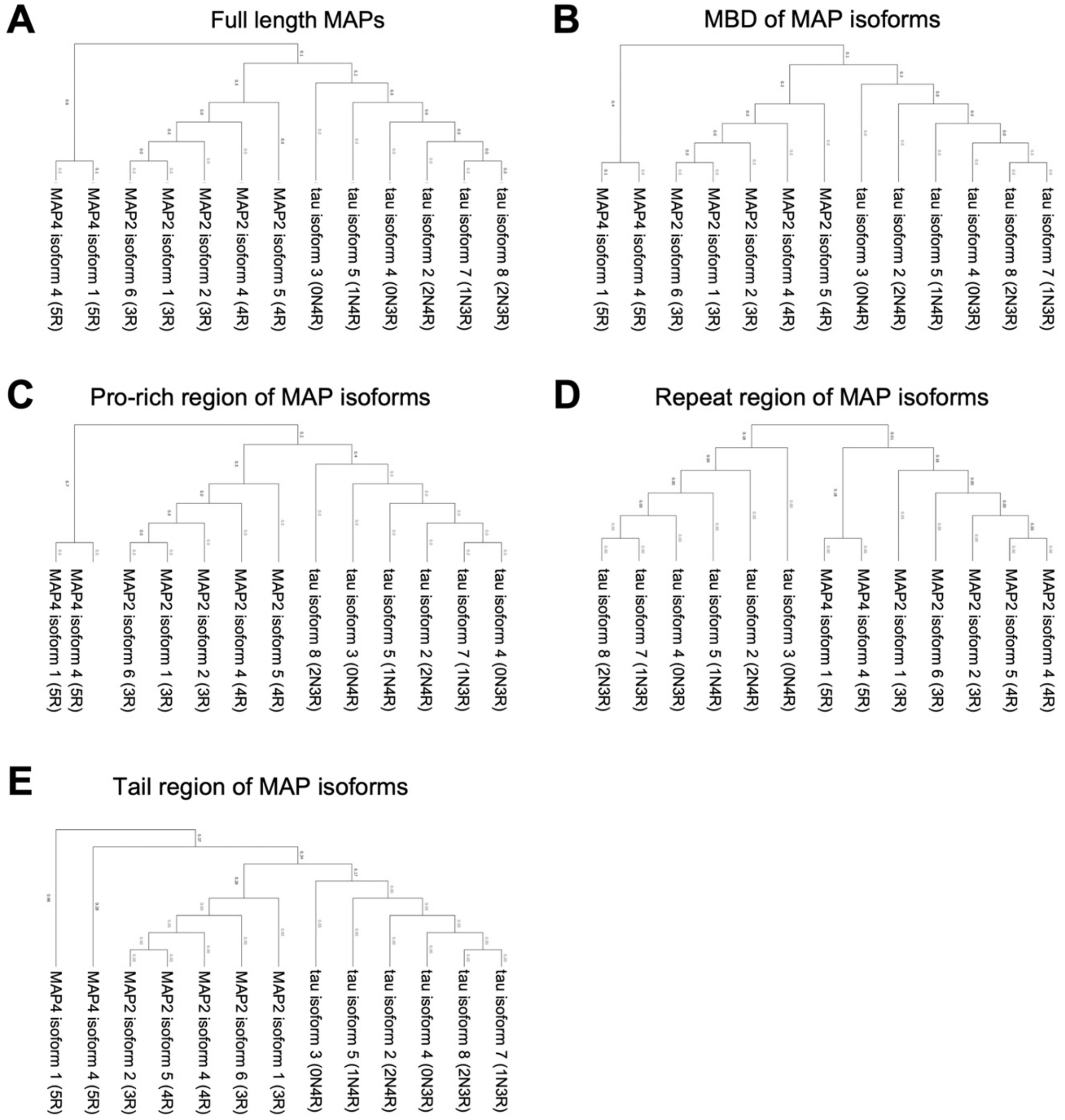

Fig. S5. Molecular phylogenetic tree analysis of human MAP isoforms. Molecular phylogenetic trees of MAP isoforms of full length (A), MBD (C), Pro-rich region (C), repeat region (D), and tail region (E). Phylogenetic trees of MAPs were created using MEGA ver. 10.2 (Masatoshi Nei, Pennsylvania State University). MUSCLE was used to align base sequences, and the neighbor-joining method was used to estimate the phylogenetic tree. The MAP isoforms used to create the phylogenetic tree are shown in Table S2. Note that only the dendrogram of the repeat region (D) shows a different pattern (and phylogenetic distance) to other dendrograms, i.e., MAP4 and MAP2 are closely related.

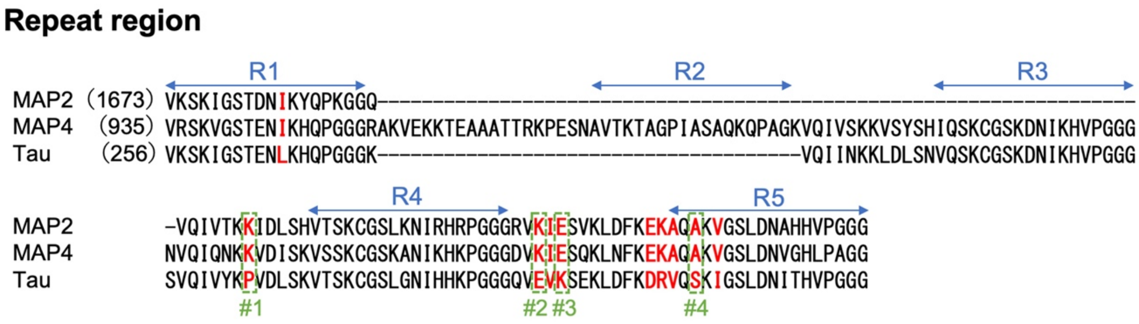

Fig. S6. Comparison of amino acid sequences of repeat regions of MAP2, MAP4, and tau, which were used in this study. R1 to R5 show the repeat sequences of MAP4. Amino acids in red indicate sites that differ only in tau, when the three MAPs are compared. The green dashed boxes indicate regions in which the amino acids of tau are significantly different from those of MAP2 and MAP4. In #1, the basic amino acid residue lysine is replaced by proline in tau. In #2, the basic amino acid residue lysine is replaced by the acidic amino acid residue glutamic acid in tau. In #3, the acidic amino acid residue glutamic acid is replaced by the basic amino acid residue lysine in tau. In #4, the hydrophobic amino acid residue alanine is replaced by the hydrophilic hydroxy amino acid residue serine in tau.

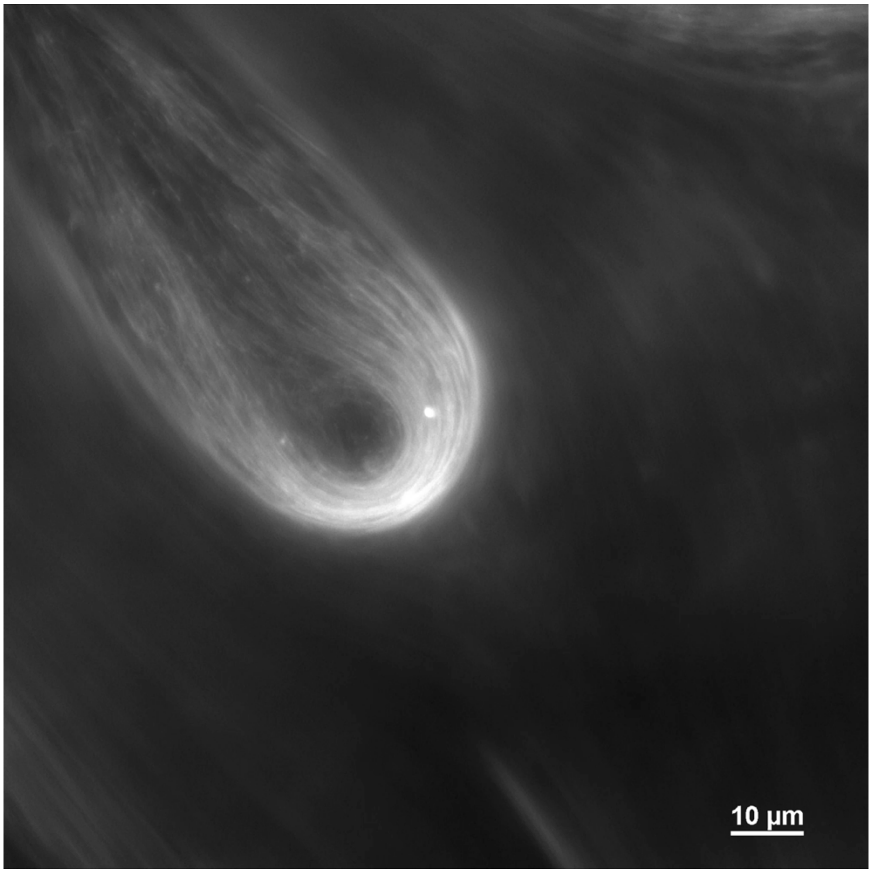

Movie S1 (separate file). Formation of a microtubule teardrop pattern. 135 μM of taxol-stabilized microtubules labeled with DyLight488 formed teardrop patterns. Fluorescence images were captured at a rate of 37 frames / 180 s.
